# Post-Traumatic, Drug-Resistant Epilepsy and Review of Seizure Control Outcomes from Blinded, Randomized Controlled Trials of Brain Stimulation Treatments for Drug-Resistant Epilepsy

**DOI:** 10.7759/cureus.744

**Published:** 2016-08-22

**Authors:** Michael Larkin, R. Michael Meyer, Nicholas S Szuflita, Meryl A Severson, Zachary T Levine

**Affiliations:** 1 School of Medicine, Uniformed Services University of the Health Sciences; 2 Department of Neurosurgery, Walter Reed National Military Medical Center/Uniformed Services University of Health Sciences; 3 Department of Neurosurgery, Holy Cross Hospital

**Keywords:** post-traumatic epilepsy, vagus nerve stimulation, deep brain stimulation, anterior nucleus thalami, responsive cortical neurostimulation, transcranial direct current stimulation, repetitive transcranial magnetic stimulation, drug-resistant epilepsy

## Abstract

Background: Many post-traumatic epilepsy (PTE) patients become resistant to medications. Nervous stimulation as a treatment for drug-resistant epilepsy (DRE) is an active area of clinical investigation.

Objective: To summarize methods, reported seizure control outcome measures, and adverse events from blinded, randomized control trials (RCTs) for selected invasive brain stimulation (IBS) and non-invasive brain stimulation (NIBS) treatment options in patients with DRE.

Methods: PubMed was searched for articles from 1995-2014, using search terms related to the topics of interest. Available relevant articles reporting the outcomes of interest were identified and data was extracted. Articles in the reference lists of relevant articles and clinicaltrials.gov were also referenced.

Results: Eleven articles were analyzed with a total of 795 patients identified. Studies showed that select nervous stimulation treatments significantly reduced seizure frequency in patients with DRE.

## Introduction and background

Epilepsy affects more than 65 million of the worldwide population and 2.2 million people in the United States [1]. In the general population, up to 20% of acquired symptomatic epilepsy results from traumatic brain injury (TBI) [2]. The risk of developing post-traumatic epilepsy (PTE) has been described as being up to a 30-50% after a TBI; a 30 times greater risk of epilepsy when compared to the general population [3-4]. Shortly after the TBI, there is a cascade of events which include contusions, cytotoxic edema, axonal shearing, inflammatory responses, free-radical generation, mitochondrial dysfunction, neurotransmitter release, and angiogenesis [5]. These changes cause an imbalance between excitatory and inhibitory processes and lead to an increased risk for the formation of spontaneous epileptic foci due to excessive excitatory stimulation [6]. Post-traumatic seizures can occur as soon as one week after injury or up to ten years or longer post-TBI [7]. For patients who have a single late seizure (>7 days TBI), more than 80% will have their second seizure within two years and will then have up to a 90% chance of progressing to PTE [7-8]. Antiepileptic drugs (AEDs) have only been demonstrated to prevent the occurrence of early seizure onset but do not prevent the late development of PTE, and their use should be discontinued after seven days if no seizures occur [9]. Currently, there are no known effective therapies to prevent PTE and no way for clinicians to accurately predict who will develop PTE. A majority of these patients will also develop drug-resistant epilepsy (DRE)--defined as a failure of an adequate trial of > 2 tolerated, appropriately chosen, and used AED regimens (monotherapy or combination) to achieve seizure cessation [10-11].

In general, AEDs result in seizure control in two-thirds of epilepsy patients, and another 7-8% of patients can be cured with surgery [5, 12]. PTE is complicated by diffuse epileptogenicity, which makes it difficult to locate areas for resection on the basis of sensor-space EEG analysis [13]. However, localization has been improved with the use of “inverse” EEG techniques that have only recently become available and are out of the scope of this review [5]. PTE is further complicated by frequently present, wide-spread encephalomalacia. The surgical removal of these damaged areas can be effective, but the inability to consistently localize seizure foci in PTE patients continues to be a barrier for complete surgical resection. Thus, there continues to be an unmet need for additional antiepileptic treatment options, as some PTE patients will not be operative candidates, and up to one-third will remain drug-resistant.

Nervous stimulation as a treatment for DRE is an active area of clinical investigation. This article will summarize the methods, reported seizure control outcome measures, and adverse events from blinded, randomized control trials (RCTs) for several invasive brain stimulation (IBS) and non-invasive brain stimulation (NIBS) therapies that have been applied to the treatment of DRE, including vagal nerve stimulation (VNS), deep brain stimulation of the anterior thalamus (DBS-ANT), responsive cortical neurostimulation (RNS), transcranial direct current stimulation (tDCS), and repetitive transcranial magnetic stimulation (rTMS). Currently, the IBS methods are the only therapies approved for clinical use. The purpose of this review is to highlight the use of the described nervous stimulation therapies for the treatment of DRE and to encourage further attention and funding be given to advance the clinical care and patient outcomes for patients presenting with post-traumatic, drug-resistant epilepsy (PTDRE). To the authors' knowledge, no such studies have been conducted.

## Review

### Materials and methods

Literature Search 

Studies were identified via a PubMed search for articles from 1995-2014, using search terms related to the treatment method: VNS, DBS, RNS, tDCS, and rTMS; and in conjunction with the keywords “seizure or epilepsy”, “frequency”, and ”decrease or reduction”. The reference lists of these articles were then examined for additional resources that fit the selection criteria. An ongoing clinical trial from clinicaltrials.gov was also included in the review. One article was discovered incidentally during the full-text review of articles.

Selection Criteria

The following criteria were utilized for article selection: (1) articles in English, (2) human trials, (3) > 12 years of age, (4) controlled trials, (5) blinded, and (6) randomized. Articles selected for full-text review were further excluded if our detailed review revealed that the above criteria were not met.

Data Extraction

The primary author (MBL) identified relevant articles reporting the outcomes of interest. Two authors (MBL, RMM) extracted relevant data independently. Each author’s data was cross-reviewed by the other, and any discrepancies, if necessary, were resolved by a third author (MAS). Standardized tables were utilized to extract the following variables: (1) seizure frequency reduction, (2) seizure frequency risk reduction, and (3) percent responders. The primary author collected the reported demographic characteristics, study design, stimulation parameters, and adverse events.

### Results

**Figure 1:**
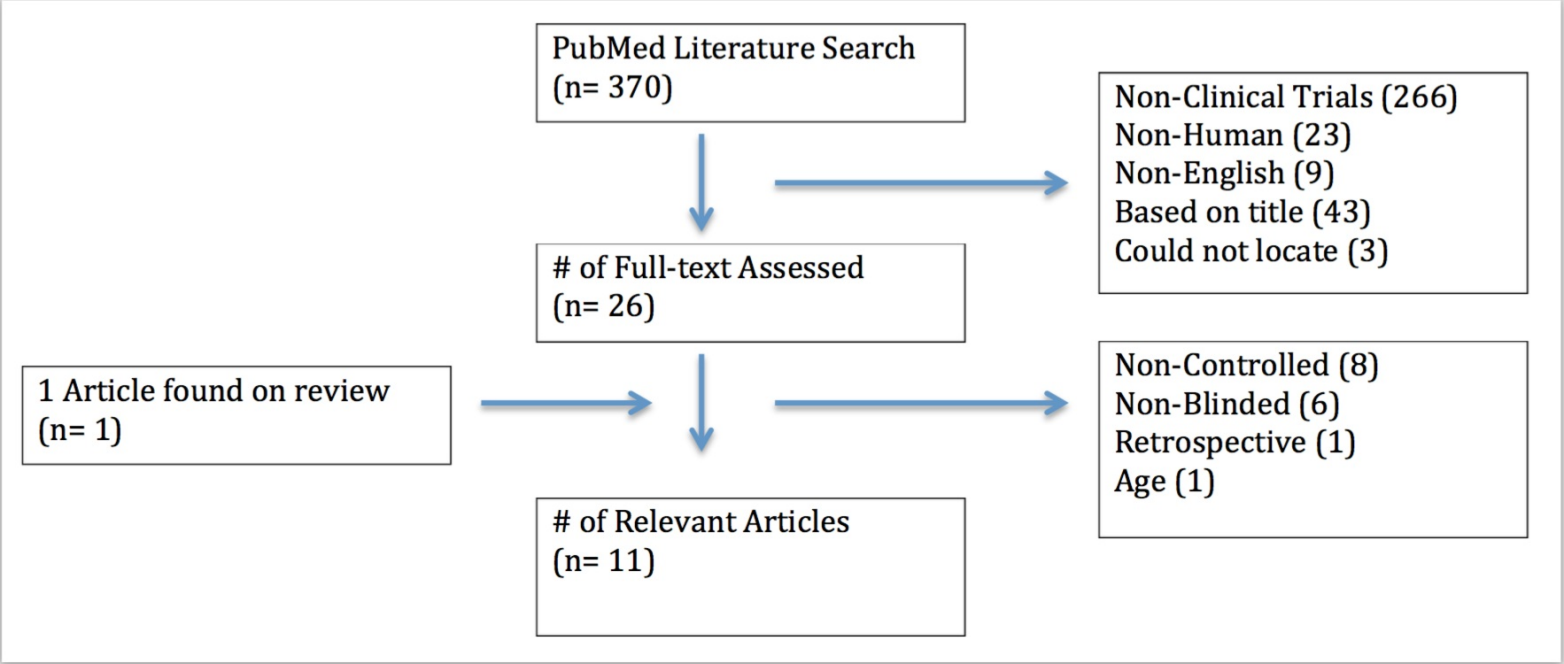
Search Flow Diagram

In total, 370 articles were identified. After applying the selection criteria, 11 relevant articles were identified with a total of total of 795 patients (Figure 1). In general, studies showed that select nervous stimulation modalities significantly reduced seizure frequency in select DRE patients.

### Discussion

Vagus Nerve Stimulation

**Figure 2:**
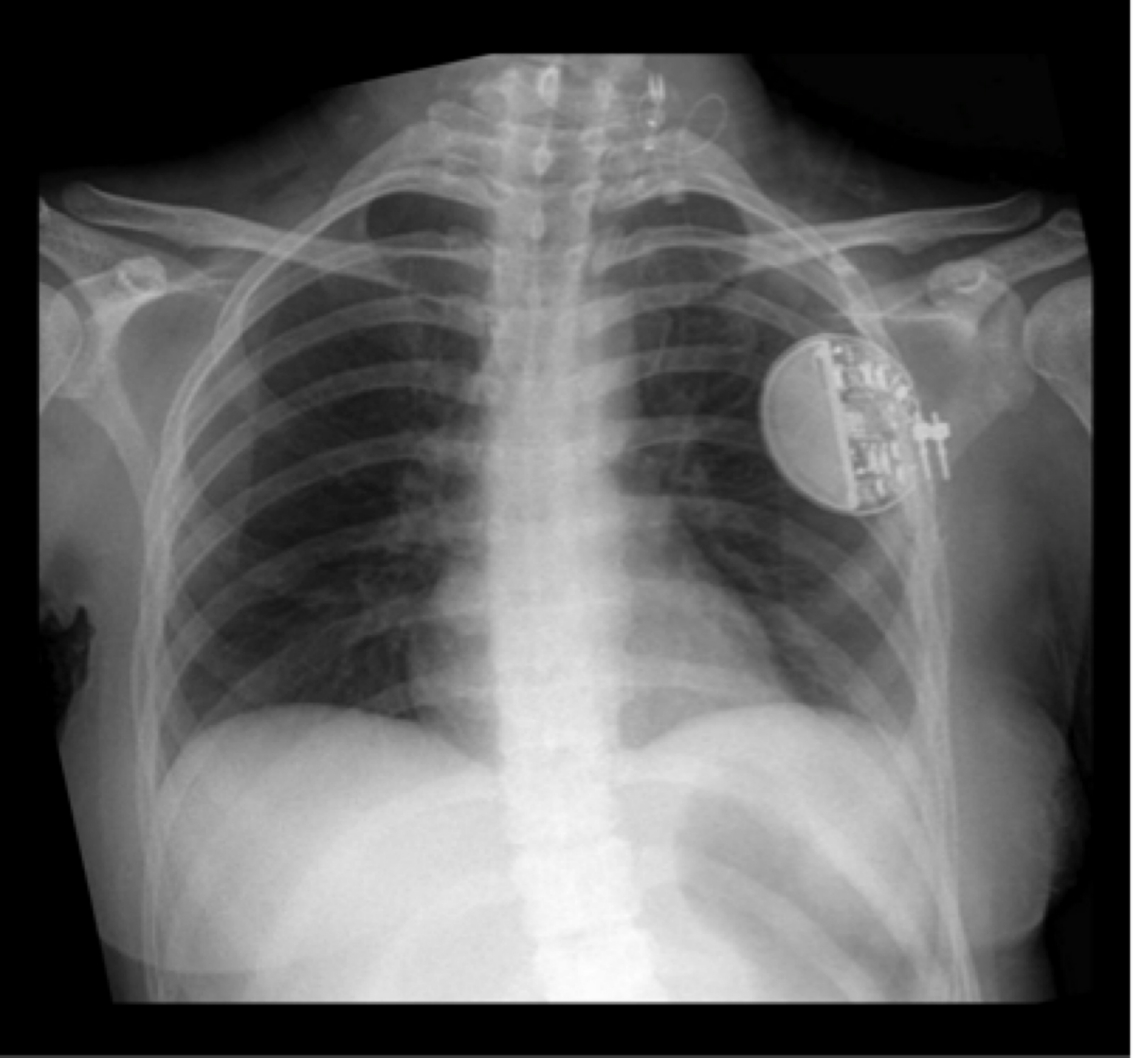
Vagal Nerve Stimulator Case courtesy of Dr. Jayanth Keshavamurthy; Radiopaedia.org, rID: 26836

In 1997, the Food and Drug Administration (FDA) approved VNS for adjunctive therapy for DRE in patients greater than twelve years old [14]. VNS devices (Figure 2) generally consist of an implantable pulse generator that is placed in the left sub-clavicular fossa with electrodes that are wrapped around the left vagus nerve distal to the take-off of the cardiac branch [15]. Typically, the right vagus nerve is avoided due to the increased potential for negative effects because of its greater role in cardiac function [16]. The exact mechanism for reduced seizure activity in VNS remains unknown. The majority of vagus nerve fibers are afferent, and the antiepileptic properties of VNS are thought to be related to these afferent effects on the brainstem reticular activating system, the central autonomic network, the limbic system, and the diffuse noradrenergic projection system; all of which have connections to numerous forebrain structures involving multiple neurotransmitters (norepinephrine and GABA) [17-20].

Three, double-blinded, randomized controlled trials have been published for the use of VNS in the treatment of DRE [15, 21-22]. Significant changes in seizure frequency from baseline in the high stimulation groups of the E03, E05, and Amar, et al. studies when compared to the low stimulation groups as well as participant demographics and stimulation parameters are summarized in Table 1. The rates of responders in the treatment groups (defined as those participants with a ≥ 50% decrease in seizure frequency) were 31%, 23.4%, and 57%, respectively.

**Table 1:** VNS Demographics and Outcomes AED: antiepileptic drugs, BEP: blinded evaluation period, %SFR: percent seizure frequency reduction, SFRR: seizure frequency risk reduction, %Resp: percent responders. *High stimulation group results.

Study	# Pts	Age (yrs)	#AEDs		Stimulation Group	Current (mA)		Pulse Time (μsec)	Freq (Hz)	ON Time (sec)	OFF Time (min)	Duration of BEP (weeks)	% SFR*		SFRR*	% Resp*
VNS																
VNSSG 1995 (E03) [15]	114	33.3	≤3	High			0.25-3.0	500	20-50	30-90	5-10	14	24.5	18.4		31
				Low			0.25-2.75	130	1-2	30	60-180					
Amar et al., 1997 [22]	17	38.8	2.6	High			≤3.5	500	30	30	5	12	71	65		57
				Low			≤3.5	130	1	30	180					
Handforth et al., 1998 (E05) [21]	196	33.1	1-3	High			<3.5	500	30	30	5	12-16	27.9	12.7		23.4
				Low			<3.5	130	1	30	180					

In the two largest trials, 352 adverse events were reported among 149 treatment group participants. The most common adverse events were current dependent and transient (occurring only during stimulus delivery), which included hoarseness, cough, and pharyngitis/throat pain accounting for 55.7% (196/352) of all reported events [15, 21]. Only one patient was found to withdraw from treatment as a result of an unspecified adverse event(s). Infections were only reported in the EO5 trial and occurred in 11.6% (23/198) of the patients [21]. However, a 2012 clinical practice guideline update endorsed by the American Epilepsy Society reported infections in only 3-7% patients [23]. In general, VNS is a low-risk procedure with minor and transient adverse post-surgical events.

Deep Brain Stimulation

**Figure 3:**
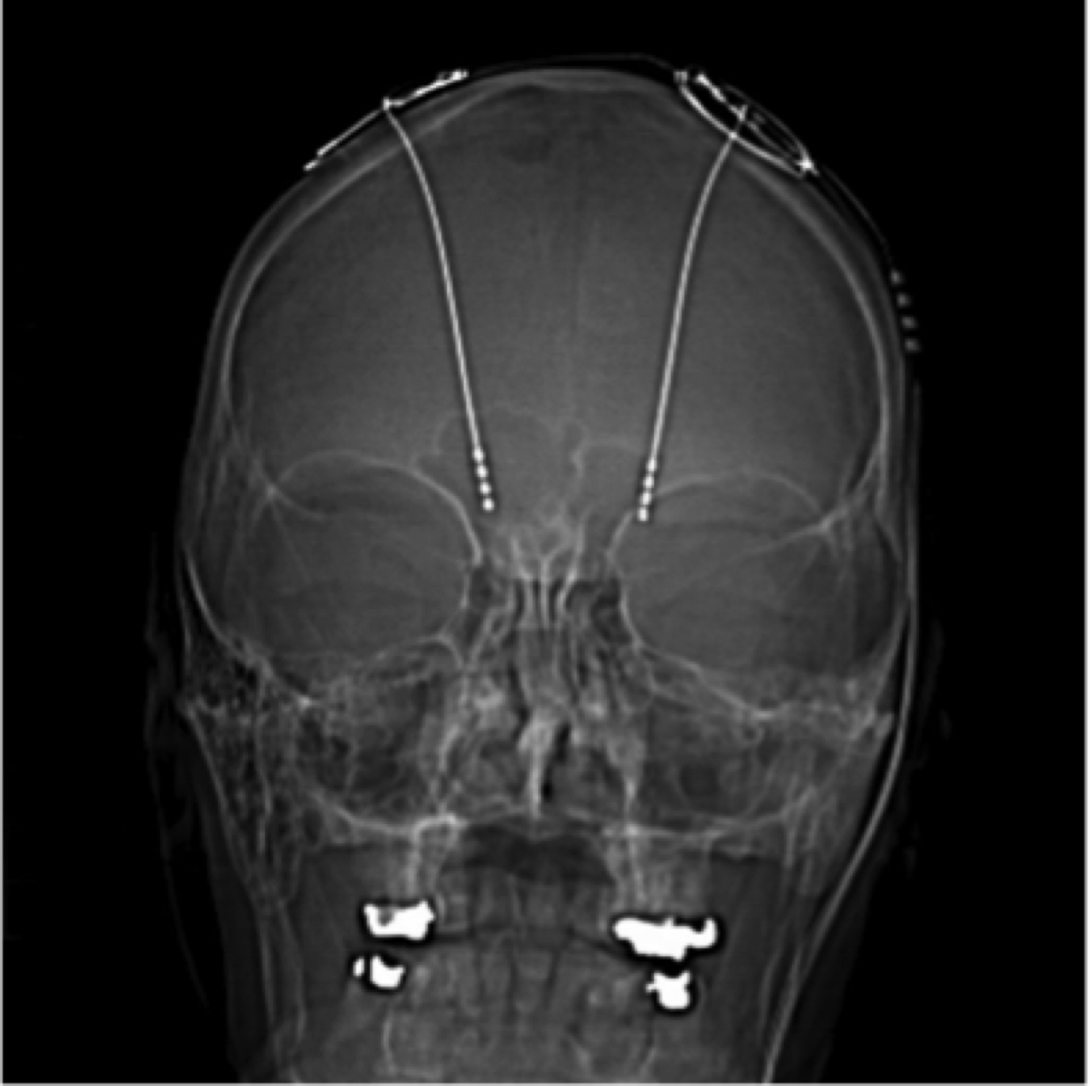
Deep Brain Stimulation Case courtesy of A.Prof Frank Gaillard; Radiopaedia.org, rID: 2743

In 1972, seizure frequency reduction was first reported with the direct implantation of stimulators in the cerebellum [24]. The mechanism of action of DBS-ANT in epilepsy is still being investigated, but authors have suggested that the reduced seizure activity with the stimulation of the anterior nuclei of the thalamus could possibly be related to its diffuse projections to the temporal lobes and its participation in the circuit of Papez [25-26] (Figure 3).

Recently, the double-blinded Stimulation of the Anterior Nucleus of the Thalamus for Epilepsy (SANTE) trial showed a significant decrease in seizure frequency for the treatment of DRE as shown in Table 2 [26]. However, the seizure reduction effect was found to be only significant in patients whose seizures originated from one or both temporal lobes but not in patients with frontal, parietal, occipital, or multifocal/diffuse seizure foci. In addition, the number of responders during the blinded evaluation period (BEP) was not significantly different between the groups [26]. In the 25-month, unblinded follow-up period, there was a statistically significant seizure frequency reduction from the baseline of 56% and a responder rate of 54% [26]. This suggests that longer-term treatment could improve outcomes to DBS-ANT in patients with DRE.

**Table 2:** DBS-ANT Demographics and Outcomes AEDs: antiepileptic drugs, BEP: blinded evaluation period, %SFR: percent seizure frequency reduction, SFRR: seizure frequency risk reduction, %Resp: percent responders. *Active stimulation group results. ^Difference between groups were not significant.

Study	# Pts	Age (yrs)	#AEDs	Stimulation Group	Voltage (V)	Pulse Time (μsec)	Freq (Hz)	ON Time (min)	OFF Time (min)	Duration of BEP (weeks)	% SFR*	SFRR*	% Resp*
DBS-ANT													
Fisher et al., 2010 [26]	110	36.1	1-4	Active	5	90	145	1	5	12	40.4	25.9	29.6^
				Sham	0	Not set in control group							

Eight hundred and eight adverse events were reported among 109 participants following surgical implantation [26]. Device-related events occurred in 29.5% (238/808); 6.8% (55/808) were considered serious as most required hospitalization [26]. The most common device-related events were paresthesias (18.2%), pain at implantation site (10.9%), and infection (9.1%) [26]. Five participants had hemorrhages that were neither symptomatic nor clinically significant and were detected incidentally. Six (5.6%) participants required complete removal of the device secondary to infection, and 18 participants (16.7%) withdrew from the trial as a result of adverse events after surgery [26].

Another potential DBS target for reducing epileptic activity is the hippocampus, also a part of the circuit of Papez. Currently, the large Controlled Randomized Stimulation Versus Resection (CoRaStir) trial is underway to assess if hippocampal stimulation will produce a greater reduction in mean monthly seizure frequency when compared to medial temporal lobe resection [27]. At the time of this review, demographics and stimulation parameters were not available to report.

Currently, DBS-ANT has been approved for use in Europe, but the FDA has not approved it for use in the United States. Should the FDA approve either DBS-ANT/hippocampal stimulation, this may provide a method for the preservation of cognitive function by allowing bilateral temporal treatment compared to patients otherwise requiring lobectomy.

Responsive Cortical Neurostimulation

**Figure 4:**
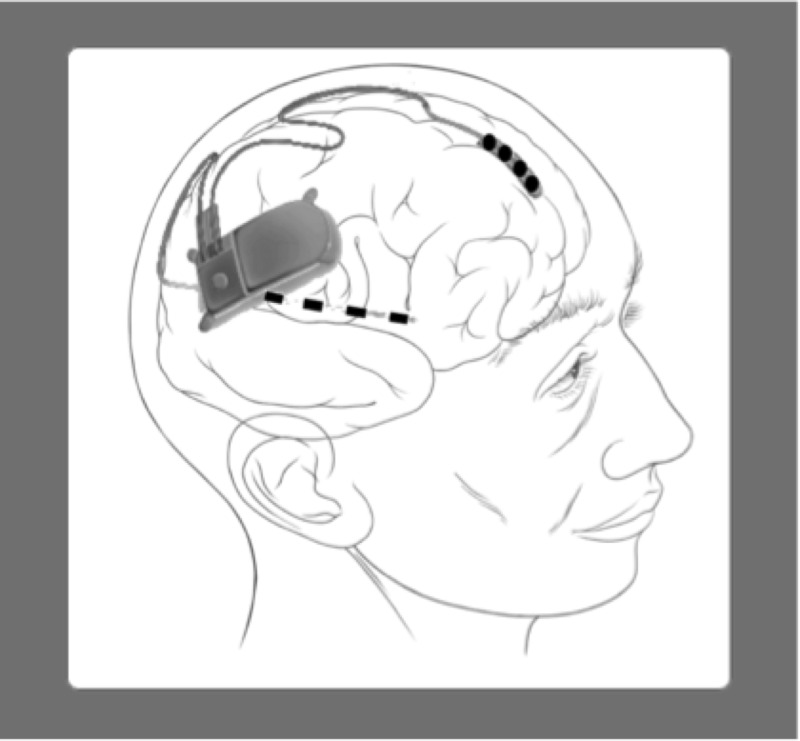
Responsive Neurostimulation

In 2013, the Neuropace® RNS® System (Neuropace, Mountain View, CA) was approved by the FDA (Figure 4). Unlike VNS, which is an open loop system that delivers a preprogrammed stimulus, the RNS system is a closed-loop system and is able to detect abnormal epileptiform activity and deliver an appropriate stimulus to prevent seizure onset [28]. Permanent subdural or depth electrodes are implanted at the seizure focus area and connected to a stimulation device embedded under the scalp. The mechanism by which RNS leads to seizure frequency reduction remains unknown, but multiple mechanisms of action are hypothesized. Acute effects are thought to include the release of neurotransmitters, local cellular inhibition/excitation, as well as cerebral blood flow changes [29]. The long-term therapeutic effects may be the result of changes in neural networks secondary to synaptic plasticity, neurogenesis/cortical reorganization [29].

The double-blinded trial data results were published separately with the initial results including the intent-to-treat analysis and first-year, open-label period (OLP) data in 2011 [30]. This was followed by a per-protocol analysis and final clinical results in 2014 [29]. Patient demographics, stimulation parameters, and seizure frequency reduction results are summarized in Table 3. There was a significant difference in seizure frequency reduction--independent of seizure onset location--between groups. There was no significant difference demonstrated in the number of responders during the initial, blinded period. However, during the OLP the median percent reduction (53%) and responder rate (55%) at two years were significantly different. Again, similar to DBS-ANT, this suggests improved efficacy with an increased duration of treatment.

**Table 3:** RNS Demographics and Outcomes AED: antiepileptic drugs, BEP: blinded evaluation period, %SFR: percent seizure frequency reduction, SFRR: seizure frequency risk reduction, %Resp: percent responders. *Active stimulation group results. **Titrated upward as tolerated to 12mA. ^Difference between groups not significant.

Study	# Pts	Age (yrs)	#AEDs	Stimulation Group	Current (mA)	Pulse Time (μsec)	Freq (Hz)	ON Time (min)	OFF Time (min)	Duration of BEP (weeks)	% SFR*	SFRR*	% Resp*
RNS													
Morrell et al., 2011 [30]	191	34.9	2.8	Active	0.5**	160	200	5.9	-	12	37.9	20.6	29^
				Sham	Off	--------Not set in control group-----							
Heck et al., 2014 [29]				Active	0.5**	160	200	5.9	-	12	41.5	32.1	-
				Sham	Off	--------Not set in control group-----							

There were 206 adverse events reported among 191 participants, with the most common being implant site pain (15.7%) and headaches (10.5%) [30]. Five participants experienced lead damage, four of which were due to complications with lead securement at the burr hole; the other was the result of inadvertent cutting and damage to a separate strip lead that was between the skull and the titanium plate. Postoperative intracranial hemorrhage occurred in 3.1% (6/191), and four of these patients required hematoma evacuation [29-30]. Postoperative, device-related implant site infections were reported in seven participants (3.7%), requiring device explantation in four patients [29-30]. The reported infections were of soft-tissue only and did not involve the brain or cranium [29-30]. Five participants withdrew from the study, none of which were the result of adverse events [29-30].

Transcranial Direct Current Stimulation

**Figure 5:**
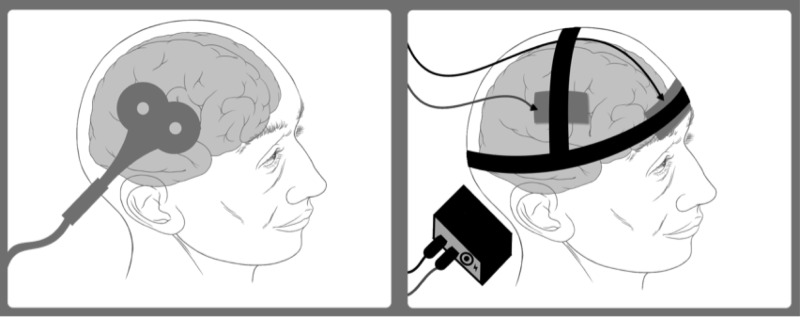
rTMS Device (Left), tDCS Device (Right)

tDCS is a non-invasive application of direct current to the scalp that subsequently causes stimulation of the cerebral cortex (Figure 5). The exact mechanism of tDCS in DRE is not completely known, but it is thought to be related to the hyperpolarization and depolarization of neurons that cause cortical inhibition at the epileptic foci with an anode-to-cathode direction of current [31]. Pharmacologic and magnetic resonance spectroscopy studies have demonstrated that the mechanism for tDCS could be the result of local neuronal effects secondary to NMDA receptor modulation, and reductions in glutamate and GABA activity [32-33].

There has been only one double-blinded RCT published; the results of which are summarized in Table 4 [34]. No statistically significant reductions in seizure frequency were found, but the treatment group showed a reduction in epileptiform discharge up to four weeks post-treatment.

**Table 4:** tDCS Demographics and Outcomes AED: antiepileptic drugs, BEP: blinded evaluation period, %SFR: percent seizure frequency reduction, SFRR: seizure frequency risk reduction, %Resp: percent responders. *Active stimulation group results. ^Difference between groups not significant.

Study	# Pts	Age (yrs)	#AEDs	Stimulus Location	Stimulation Group	Current (mA)	ON Time	Duration of BEP	% SFR*	SFRR*	% Resp
tDCS											
Fregni et al., 2006 [34]	19	24.2	2.3	Seizure Focus	Active	1	20 min	30 days	44^	32.9	-
					Sham	1	30 sec				

Adverse events with tDCS are minimal as a limited number of minor adverse events reported at the site of stimulation; four patients (21.1%) reported mild itching [34].

Currently, tDCS is not FDA approved for clinical use. The current data for tDCS may support the idea that it may be safe, but is insufficient to make any clinically relevant conclusions regarding its therapeutic use for DRE, and further studies are needed. 

Transcranial Magnetic Stimulation

rTMS is a repetitive, electrical, non-invasive brain stimulation (NIBS) method that has been shown to be effective in some psychiatric and neurologic disorders (Figure 5). In rTMS, an alternating current is run through a wire coil to create a fluctuating magnetic field that is then focused on the area of interest in the brain, inducing intracranial electrical currents [35]. Excitation of the cortex is enhanced by high-frequency stimulation (>1Hz), while low stimulation rates (≤1Hz) reduce cortical excitation [36-37]. Different coils are designed to stimulate either superficial and smaller foci or deeper and broader foci [38]. The exact mechanism of action is still unknown. It is thought to be related to the currents generated by the magnetic field, which lead to changes in the concentration of neurotransmitters (primarily glutamate and dopamine), and has been shown to be related to stimulation intensity and frequency delivered [36, 39-40]. Clinical effects of rTMS are related to the temporal spacing and duration of the stimulation, and it is believed that induced cortical plasticity may be the result of NMDA receptor modulation [41-42].

The use of rTMS as a non-invasive, antiepileptic therapeutic option for patients with DRE has had mixed results based on the four published, randomized controlled trials summarized in Table 5. In two trials--Theodore et al., and Cantello et al.,--no significant differences in seizure frequency reduction were found [43-44]. In contrast, two studies have demonstrated significant reductions in seizure frequency in patients with a predetermined location of cortical seizure foci [41, 45]. Both of these studies included patients with cortical dysplasia or superficial epilepsy origin, which had been excluded in previous trials [43]. A significant decrease in seizure frequency, eight weeks after stimulation, was demonstrated suggesting that the initial rTMS treatment had a longer-lasting effect on seizure frequency reduction, even after the scheduled treatment was completed. Additionally, Sun et al., (a single-blinded trial) demonstrated that 35.5% of patients remained seizure free, and 22.6% of patients had a complete abolishment of epileptiform discharges at the end of the blinded evaluation period (BEP).

**Table 5:** rTMS Demographics and Outcomes AED: antiepileptic drugs, BEP: blinded evaluation period, %SFR: percent seizure frequency reduction, SFRR: seizure frequency risk reduction, %Resp: percent responders, MO: maximum output, RMT: resting motor threshold, BID: twice daily, QD: daily. *Active stimulation group results or High Stimulation Group. ^Difference between groups not significant.

Study	# Pts	Age (yrs)	#AEDs	Coil Type	Stimulus Location	Group	Strength	Freq (Hz)	#Pulses	Schedule	Duration of BEP (weeks)	% SFR*	SFRR*	% Resp*
rTMS														
Theodore et al., 2002 [43]	24	40	-	Figure 8	Seizure Focus	Active	120% RMT	1	900	BID for 1 week	9	4.5^	4.9	-
Fregni et al., 2006 [41]	21	21.9	2.2	Figure 8	Seizure Focus	Active	70% MO	1	1200	QD for 5 days	9	58	-	83
Cantello et al., 2007 [44]	43	36.9	2 to 4	Round	Vertex	Active	100% RMT	0.3	2 x 500; 30 sec apart	QD for 5 days	7	13.2^	3.3	14.7
Sun et al., 2012 [45]	60	20.5	1 to ≥3	Figure 8	Seizure Focus	High	90% RMT	0.5	3 x 500; 600 sec apart	QD for 2 weeks	10	79.8	77.5	-
						Low	20% RMT	0.5	3 x 500; 600 sec apart	QD for 2 weeks				

Other authors have noted that the reason for the non-significant results for Theodore et al., may have been secondary to their recruitment of subjects with deep epileptic origin [39]. This results in a decreased applied magnetic field whose strength is a cubic function of the distance between the rTMS device and the seizure foci being stimulated [43]. A greater, but still non-significant, improvement was observed among study participants who had a more lateral temporal seizure foci compared to those with deeper mesial temporal foci [39, 43].

The Cantello, et al. trial randomized patients to treatment with stimulation from a circular coil, based on previous reports of efficacy [44]. The authors attributed their poor results to the unpredictable variability in seizure frequency in relation to their sample size [44]. However, round coils have been shown to provide a more diffuse and deep effect compared to the focused and superficial effects seen with figure-eight coils [38]. For successful application of rTMS in the treatment of DRE, stimulation at the exact location of the seizure focus is critical [41]. This explanation offers a better reason for the lack of significant seizure reduction difference between groups in this particular study.

Among all rTMS trials reviewed, the vast majority of adverse events were minor, occurring in 19 (12.8%) of the collective 148 participants [41, 43–45]. The most commonly reported events included headache, dizziness, and tinnitus. One patient on two occasions experienced a typical complex partial seizure, and as a result did not complete the entire week of stimulation but remained a part of the trial [43]. Patients should be cautioned that rTMS could induce seizures in those who are already seizure-prone versus non-epileptic individuals. However, a literature review of rTMS in 280 DRE patients found only four patients who had a seizure during treatment, only one of whom had an atypical seizure whose focus was different from their previously noted seizure origin. Importantly, this event occurred in a participant who was receiving a high-frequency (16Hz) stimulation, which has been shown to cause cortical excitation [46]. The risk of additional seizures with rTMS stimulation in patients with DRE was nearly as safe to those without epilepsy [46].

### Primary outcome reporting

All studies included in this review reported mean/median seizure frequency reduction as the primary outcome and measure of clinical improvement. However, in some trials no significant differences were found in the reported number of percent responders, those participants with a ≥ 50% seizure frequency reduction, suggesting that a majority of patients who benefited during this time may have had only modest results [26, 30]. A reasonable alternative might be to report percent responders as the primary outcome. This would correlate better to consecutive seizure-free days in patients. Additionally, reporting of seizure frequency risk reduction (SFRR)--synonymous with absolute risk reduction, calculated as the difference in seizure frequency between both groups--would allow reporting of the number needed to treat (NNT). The same method could be applied to percent responders. The authors suggest that increasing the reporting of SFRR and percent responder data would improve the quality of clinically relevant outcome data available.

### Limitations of non-invasive stimulation studies

Sham Protocols

The design of nervous stimulation trials, both IBS and NIBS, can be complicated by the difficulty of reproducing sham conditions that replicate active treatment and maintain participant blinding in order to minimize placebo effect. A reliable sham protocol for tDCS has been described as giving a short (30 sec) stimulus, which gives the initial sensation of treatment but maintains blinding because of the fast attenuation of the stimulus in those with a sustained stimulation [47]. Sham is a much greater concern in rTMS trials because the stimulation is accompanied by a loud sound, with strong scalp sensations and muscle twitching. Thus, patients can become unblinded during titration of the resting membrane threshold (RMT). Fregni, et al. based their stimulation rTMS protocol on the maximal output of the device and the use of a replica that produced a similar sound artifact, which required no titration to RMT avoiding this limitation [41]. Other rTMS studies have utilized sham procedures that included rotation or increased distance of the device from the scalp [43-44]. Sun et al., used a low-intensity RMT stimulus protocol for sham, and neither group received 100% RMT. This suggests blinding may have been preserved. However, there was no discussion of the differences, if any, in sensations among the groups. The use of focal electrical stimulation and personalized titration to simulate rTMS stimulation has also been investigated and described in a small study as a possible alternative sham protocol, but further validation is needed [48]. Nonetheless, a measure of the sham effect for three out of the four rTMS trials in this review was shown to have a minimal effect on seizure frequency reduction and responder rates [49]. The use of “active control groups” (in VNS and rTMS) differs from truly blinded patients, and this difference in data quality can affect the comparison of IBS and NIBS protocols. Future RCTs for nervous stimulation should include participants who are naïve to therapy, crossover study designs should be avoided, and care should be taken to ensure participants receive therapy separately from other study participants.

Stimulation Variability

The standards for stimulation parameters between brain stimulation trials are very heterogeneous with respect to applied current, frequency, strength, duration, and the number of treatment sessions. Ziemann, et al. discussed the further complicating issue of inter-subject and inter-session variability of plasticity induction among NIBS studies and suggest that researchers design experimental and stimulation-priming protocols that are carefully standardized by collecting EEG recordings during stimulation [50]. This would allow for the continued investigation and determination of the underlying causes of inter-session and inter-subject variability in order to develop techniques to limit such variability and improve clinical efficacy.

## Conclusions

Despite improvements, the localization of surgically amendable seizure foci remains difficult due to diffuse epileptogenic foci and the presence of encephalomalacia in post-traumatic DRE (PTDRE) patients. For affected service members, this makes identifying alternative therapies that are efficacious a worthy undertaking. To the authors' knowledge, no such studies have been conducted specifically investigating the efficacy of nervous stimulation in the PTDRE population. As described in detail above and summarized in Table 6, the evidence for the efficacy of NIBS for DRE in the general population has been growing, but primary outcome results are still far less convincing than that seen with IBS or surgical resection in eligible patients. The use of tDCS and rTMS for the treatment of DRE are still investigational, and there are currently no FDA-approved indications for their use. The IBS therapies for the treatment of DRE have been shown in multiple, large, blinded, randomized controlled trials to be effective options for reducing seizure frequency and are currently in clinical use. The authors’ hope is that this review encourages future funding for research investigating the role of NIBS and IBS therapies for PTDRE. Research and treatments should focus on improving data reporting, improving sham protocols, and reducing stimulation response variability in order to determine practical and clinically effective alternative treatment options where surgery is either not an option, has failed, or for patients who prefer a non-invasive approach.

**Table 6:** Summary %SFR: percent seizure frequency reduction, SFRR: seizure frequency risk reduction, %Resp: percent responders. *Active stimulation group results. ^Difference between groups not significant.

Study	FDA Approved	% SFR*	SFRR*	% Resp*
VNSSG 1995 (E03) [15]	Y	24.5	18.4	31
Amar et al., 1997 [22]	Y	71	65	57
Handforth et al., 1998 (E05) [21]	Y	27.9	12.7	23.4
Fisher et al., 2010 (SANTE) [26]	N	40.4	25.9	29.6^
Morrell et al., 2011 [30]	Y	37.9	20.6	29^
Heck et al., 2014 [29]	Y	41.5	32.1	-
Fregni et al., 2006 [34]	N	44	32.9	-
Theodore et al., 2002 [43]	N	4.5^	4.9	-
Fregni et al., 2006 [41]	N	58	-	83
Cantello et al., 2007 [44]	N	13.2^	3.3	14.7
Sun et al., 2012 [45]	N	79.8	77.5	-

## References

[1] Committee on the Public Health Dimensions of the Epilepsies, Board on Health Sciences Policy, Institute of Medicine of the National Academies (2012). Epilepsy Across the Spectrum: Promoting Health and Understanding. National Academy of Sciences.

[2] Annegers JF, Hauser WA, Coan SP, Rocca WA (1998). A population-based study of seizures after traumatic brain injuries. N Engl J Med.

[3] Diaz-Arrastia R, Agostini MA, Madden CJ, Van Ness PC (2009). Posttraumatic epilepsy: the endophenotypes of a human model of epileptogenesis. Epilepsia.

[4] Lowenstein DH (2009). Epilepsy after head injury: an overview. Epilepsia.

[5] Irimia A, Van Horn JD (2015). Epileptogenic focus localization in treatment-resistant post-traumatic epilepsy. J Clin Neurosci.

[6] Dudek FE, Sutula TP (2007). Epileptogenesis in the dentate gyrus: a critical perspective. Prog Brain Res.

[7] Haltiner AM, Temkin NR, Dikmen SS (1997). Risk of seizure recurrence after the first late posttraumatic seizure. Arch Phys Med Rehabil.

[8] Hess CP, Barkovich AJ (2010). Seizures: emergency neuroimaging. Neuroimaging Clin N Am.

[9] Brain Trauma Foundation, American Association of Neurological Surgeons, Congress of Neurological Surgeons (2007). XIII. Antiseizure prophylaxis. J Neurotrauma.

[10] Kwan P, Schachter SC, Brodie MJ (2011). Drug-resistant epilepsy. N Engl J Med.

[11] Semah F, Picot MC, Adam C (1998). Is the underlying cause of epilepsy a major prognostic factor for recurrence?. Neurology.

[12] Litt B, Echauz J (2002). Prediction of epileptic seizures. Lancet Neurol.

[13] Schuele SU, Lüders HO (2008). Intractable epilepsy: management and therapeutic alternatives. Lancet Neurol.

[14] (2014). Medical Devices, VNS Therapy System - P970003s050. VNS Ther.

[15] The Vagus Nerve Stimulation Study Group (1995). A randomized controlled trial of chronic vagus nerve stimulation for treatment of medically intractable seizures. Neurology.

[16] Rutecki P (1990). Anatomical physiological, and theoretical basis for the antiepileptic effect of vagus nerve stimulation. Epilepsia.

[17] McLachlan RS (1993). Suppression of interictal spikes and seizures by stimulation of the vagus nerve. Epilepsia.

[18] Krahl SE, Clark KB, Smith DC, Browning RA (1998). Locus coeruleus lesions suppress the seizure-attenuating effects of vagus nerve stimulation. Epilepsia.

[19] Ben-Menachem E, Hamberger A, Hedner T (1995). Effects of vagus nerve stimulation on amino acids and other metabolites in the CSF of patients with partial seizures. Epilepsy Res.

[20] Henry TR (2002). Therapeutic mechanisms of vagus nerve stimulation. Neurology.

[21] Handforth A, DeGiorgio CM, Schachter SC (1998). Vagus nerve stimulation therapy for partial-onset seizures: a randomized active-control trial. Neurology.

[22] Amar AP, Heck CN, Levy ML (1998). An institutional experience with cervical vagus nerve trunk stimulation for medically refractory epilepsy: rationale, technique, and outcome. Neurosurgery.

[23] Morris GL 3rd, Gloss D, Buchhalter J, Mack KJ, Nickels K, Harden C (2013). Evidence-based guideline update: vagus nerve stimulation for the treatment of epilepsy: report of the guideline development subcommittee of the american academy of neurology. Epilepsy Curr.

[24] Cooper IS, Amin I, Gilman S (1973). The effect of chronic cerebellar stimulation upon epilepsy in man. Trans Am Neurol Assoc.

[25] Fisher RS (2013). Deep brain stimulation for epilepsy. Handb Clin Neurol.

[26] Fisher R, Salanova V, Witt T (2010). Electrical stimulation of the anterior nucleus of thalamus for treatment of refractory epilepsy. Epilepsia.

[27] (2014). Controlled Randomized Stimulation Versus Resection (CoRaStiR). http://clinicaltrials.gov/ct2/show/NCT00431457.

[28] Morrell M (2006). Brain stimulation for epilepsy: can scheduled or responsive neurostimulation stop seizures?. Curr Opin Neurol.

[29] Heck CN, King-Stephens D, Massey AD (2014). Two-year seizure reduction in adults with medically intractable partial onset epilepsy treated with responsive neurostimulation: final results of the RNS System Pivotal trial. Epilepsia.

[30] Morrell MJ, RNS System in Epilepsy Study Group (2011). Responsive cortical stimulation for the treatment of medically intractable partial epilepsy. Neurology.

[31] Kabakov AY, Muller PA, Pascual-Leone A, Jensen FE, Rotenberg A (2012). Contribution of axonal orientation to pathway-dependent modulation of excitatory transmission by direct current stimulation in isolated rat hippocampus. J Neurophysiol.

[32] Nitsche MA, Fricke K, Henschke U (2003). Pharmacological modulation of cortical excitability shifts induced by transcranial direct current stimulation in humans. J Physiol.

[33] Stagg CJ, Best JG, Stephenson MC (2009). Polarity-sensitive modulation of cortical neurotransmitters by transcranial stimulation. J Neurosci.

[34] Fregni F, Thome-Souza S, Nitsche MA, Freedman SD, Valente KD, Pascual-Leone A (2006). A controlled clinical trial of cathodal DC polarization in patients with refractory epilepsy. Epilepsia.

[35] Hallett M (2000). Transcranial magnetic stimulation and the human brain. Nature.

[36] Chen R, Classen J, Gerloff C (1997). Depression of motor cortex excitability by low-frequency transcranial magnetic stimulation. Neurology.

[37] Maeda F, Keenan JP, Tormos JM, Topka H, Pascual-Leone A (2000). Modulation of corticospinal excitability by repetitive transcranial magnetic stimulation. Clin Neurophysiol.

[38] Rossi S, Hallett M, Rossini PM, Pascual-Leone A, Safety of TMS Consensus Group (2009). Safety, ethical considerations, and application guidelines for the use of transcranial magnetic stimulation in clinical practice and research. Clin Neurophysiol.

[39] Hemond CC, Fregni F (2007). Transcranial magnetic stimulation in neurology: what we have learned from randomized controlled studies. Neuromodulation.

[40] Pascual-Leone A, Amedi A, Fregni F, Merabet LB (2005). The plastic human brain cortex. Annu Rev Neurosci.

[41] Fregni F, Otachi PT, Do Valle A (2006). A randomized clinical trial of repetitive transcranial magnetic stimulation in patients with refractory epilepsy. Ann Neurol.

[42] Joo EY, Han SJ, Chung SH, Cho JW, Seo DW, Hong SB (2007). Antiepileptic effects of low-frequency repetitive transcranial magnetic stimulation by different stimulation durations and locations. Clin Neurophysiol.

[43] Theodore WH, Hunter K, Chen R (2002). Transcranial magnetic stimulation for the treatment of seizures: A controlled study. Neurology.

[44] Cantello R, Rossi S, Varrasi C (2007). Slow repetitive TMS for drug-resistant epilepsy: clinical and EEG findings of a placebo-controlled trial. Epilepsia.

[45] Sun W, Mao W, Meng X, Wang D, Qiao L, Tao W (2012). Low-frequency repetitive transcranial magnetic stimulation for the treatment of refractory partial epilepsy: a controlled clinical study. Epilepsia.

[46] Bae EH, Schrader LM, Machii K (2007). Safety and tolerability of repetitive transcranial magnetic stimulation in patients with epilepsy: a review of the literature. Epilepsy Behav.

[47] Gandiga PC, Hummel FC, Cohen LG (2006). Transcranial DC stimulation (tDCS): A tool for double-blind sham-controlled clinical studies in brain stimulation. Clin Neurophysiol.

[48] Arana AB, Borckardt JJ, Ricci R (2008). Focal electrical stimulation as a sham control for repetitive transcranial magnetic stimulation: Does it truly mimic the cutaneous sensation and pain of active prefrontal repetitive transcranial magnetic stimulation?. Brain Stimul.

[49] Bae EH, Theodore WH, Fregni F, Cantello R, Pascual-Leone A, Rotenberg A (2011). An estimate of placebo effect of repetitive transcranial magnetic stimulation in epilepsy. Epilepsy Behav.

[50] Ziemann U, Siebner HR (2015). Inter-subject and inter-session variability of plasticity induction by non-invasive brain stimulation: boon or bane?. Brain Stimul.

